# Revealing Relationships Between Academic Support Services Utilization and Step 1 Outcomes: A Structural Equation Modeling Investigation

**DOI:** 10.1007/s40670-026-02811-7

**Published:** 2026-07-01

**Authors:** Paul Sturgis, Nicole Brooks, Carley Blades Myszkowski, Jonathan Kibble

**Affiliations:** 1 College of Medicine, University of Central Florida, Orlando, FL USA; 2 Knowledge Management and Analytics, University of Central Florida College of Medicine, Orlando, FL 32827 USA

**Keywords:** Academic Support, Tutoring, Coaching, Advising, Structural Equation Modeling

## Abstract

Medical schools are increasingly adopting proactive academic support models, yet evidence on how students use these services and whether they are effective remains limited. This study examined Student Academic Support Services (SASS) use at one U.S. medical school and its relationship to United States Medical Licensing Examination (USMLE) Step 1 outcomes using records from 114 students in the Class of 2024. Students used SASS at varying levels, ranging from no visits to more than 10 during the preclerkship years; 14 students (12%) had documented disability accommodations. Students with lower academic performance—based on pre-admission measures (MCAT, GPA) and course exam results—were more likely to use SASS. In regression analyses, course exam performance was positively associated with Step 1 success, while SASS use was inversely associated when examined separately but not when included in the same model. Structural equation modeling clarified these relationships, demonstrating that both poorer course performance and higher SASS use were associated with an increased risk of Step 1 failure, while MCAT scores and undergraduate GPA exerted indirect effects. Disability accommodation status was not associated with Step 1 outcomes and did not improve model fit. These findings suggest that students with prior standardized test and academic difficulty are more likely to seek SASS support, rather than support use itself contributing to poorer outcomes. The study also demonstrates the value of advanced analytic approaches for understanding complex, interrelated predictors. Future research should replicate these findings in larger, multi-institutional samples and more directly evaluate the impact of academic support services.

## Introduction

Over the past two decades, models of medical student support have shifted from reactive systems focused on identifying and remediating “at risk” learners to more proactive, developmental, and growth oriented approaches [1, 2]. In parallel, the widespread adoption of competency‑based medical education [3], with its emphasis on individualized learner development and the cultivation of master adaptive learners [4], has further amplified the need for robust, multimodal systems of student support and coaching. Although the medical education literature provides thoughtful theoretical and philosophical analyses of coaching and support models, a persistent concern is the relative scarcity of primary evidence evaluating their impact [1, 2, 5]. The first goal of the present study was therefore to quantitatively evaluate overall utilization patterns and outcomes associated with our Student Academic Support Services (SASS) office.

An important subgroup of students utilizing academic support services includes those with formal accommodations for a learning disability, a group with unique needs that typically represents approximately 4–5% of the national medical student population [6]. Several studies have examined whether students with disabilities are at risk for lower academic performance, though findings remain inconsistent, with examples of both equivalent [7] and lower performance [8] depending on the domain assessed. The second goal of this study was to investigate patterns of SASS utilization and United States Medical Licensing Examination (USMLE) Step 1 outcomes among students with disabilities in our cohort.

A methodological limitation common to studies predicting educational outcomes is the presence of substantial correlations among predictor variables. This phenomenon is often described as multicollinearity and may also contribute to confounding when variables are related to one another and to the outcome of interest. In such cases, shared variance among predictors can obscure the independent contribution of individual variables, thereby complicating interpretation of model estimates. Structural equation modeling (SEM) offers a key advantage over traditional regression approaches because it can appropriately model correlated predictors rather than assuming independence among them [9]. SEM represents a family of advanced analytic techniques that build upon correlation, regression, and analysis of variance (ANOVA). It enables systematic examination of multivariate relationships, including interconnections among measured and latent constructs, and supports the development and testing of conceptual models [10–12]. By integrating the measurement strengths of factor analysis with the relational insights of path analysis, SEM allows researchers to evaluate both direct and indirect effects among variables, while also providing indices of model fit to assess the validity of proposed pathways. The final goal of the present study was to compare findings derived from regression and SEM analyses to illustrate the added value of SEM when collinearity among variables is present.

## Materials and Methods

### Educational Setting

The University of Central Florida (UCF) College of Medicine offers a four‑year M.D. program enrolling 120 students annually. The first two years constitute the preclerkship period, during which students complete an integrated, systems‑based basic science curriculum that incorporates clinical medicine and health‑systems science throughout. At the end of the second year, students are required to pass the USMLE Step 1 before progressing to third‑year core clerkships. In the current study, we focused on usage of academic support services during the preclerkship period and used students’ pass/fail status on the USMLE Step 1 as the dependent outcome variable in our analyses. The clinical curriculum in years 3 and 4 consists of the core clerkships throughout year 3 and advanced electives in year 4. Students typically take the USMLE Step 2 Clinical Knowledge (CK) examination during the transition between the third and fourth years.

### Student Academic Support Services (SASS)

The SASS office, housed within the Office of Student Affairs, has two full-time learning specialists and serves as the central hub for academic skill development and individualized support for medical students across all four years. SASS provides one-on-one academic coaching, small group advising, and large‑group sessions focused on study strategies, time management, test-taking skills, and preparation for major assessments such as USMLE Step 1 and Step 2 CK. The office also delivers required Step 1 workshops, individualized study planning meetings for all M2 students, and targeted outreach at key curricular transition points. In addition to professional staff support, SASS oversees the Peer Academic Coaching (PAC) program, which offers free peer tutoring, group review sessions, and student led workshops staffed by trained, high‑performing M2–M4 tutors. All services are available in person, virtually, or through recorded sessions.

Students arrive at SASS through multiple pathways that ensure broad accessibility and early intervention. Access may occur through self referral, faculty referral, or outreach from SASS after monitoring student performance on early summative assessments, with the earliest identification occurring after the first M1 summative exam. Students are informed of SASS resources through orientation, class-wide emails, presentations, and the College of Medicine website, ensuring consistent awareness and seamless access to academic support throughout the curriculum.

### Medical Students Accessibility Services

The SASS office also serves as the COM Medical Student Accessibility Liaison and manages all requests for disability accommodations within the MD program, in compliance with the Americans with Disabilities Act. The SASS office coordinates and proctors assessments for students with approved testing accommodations.

### Study Design

This was a retrospective cohort study using historical student records from the UCF COM Class of 2024. We implemented a two phase analytic design: (1) a correlation and binary logistic regression phase to estimate associations between SASS office consultations and Step 1 pass/fail, sequentially adjusting for pre admission (MCAT, undergraduate GPA) and in program performance (final course exam scores); and (2) a structural equation modeling (SEM) phase to account for correlated predictors and to estimate the direct effects of SASS consultations and course exam performance on Step 1 outcomes, while incorporating MCAT and undergraduate GPA as upstream correlates. A subgroup analysis was also conducted to investigate the outcomes for students with disability accommodations in terms of their SASS utilization and Step 1 outcomes. The study, which utilized de-identified data provided by the SASS office, was classified as Not Human Subjects Research by the UCF Institutional Review Board (study # 00008486).

### Phase 1: Correlation and Regression

The dependent variable examined in the analysis was passing or failing the Step 1 exam. Independent variables (summarized in Table 1) included undergraduate GPA, MCAT score, cumulative final exam performance across preclinical courses, and the number of consultations with the SASS office during the first two years of medical school. Demographic variables included gender (female as the reference category), and race (non-White as the reference category). Additionally, disability accommodation status, with students without a disability accommodation being the reference category, was also used as an independent variable.

**Table 1:** Descriptive statistics of study participants including demographic characteristics, academic performance metrics, student support utilization, and Step 1 performance (n = 114)

	n	%	Min	Max	Mean	SD
Step 1 Outcome (Pass)	106	93.0	—	—	—	—
Male (Yes)	58	50.9	—	—	—	—
Disability Accommodation (Yes)	14	12.3	—	—	—	—
MCAT Score	—	—	508	523	513.45	2.82
Undergraduate GPA	—	—	3.26	4.00	3.81	0.18
Total Exam Score	—	—	618.61	879.89	775.92	53.75
Number of Support Consultations	—	—	0	16	2.70	3.17

The purpose of the correlation analysis was twofold. It was utilized to determine which variables should be included in the regression analysis. Additionally, the results of both the correlation and regression analyses were used to establish the direction and significance of the relationships between the variables. This information was used to construct the model that was tested with SEM.

Binary logistic regression was selected as an analysis technique as the dependent variable, Step 1 performance, is binary, as students either passed or failed the exam. As discussed below, we ran a number of different regression models in order to elucidate how the variables were related.

#### Phase 2: Structural Equation Modeling

Conducting a SEM analysis is an iterative, five step process:

1. **Model specification**: In this step, the researcher identifies the variables that will be utilized in the analysis, and specifies how the variables in the model are related. The model should be based upon previous empirical findings, or a strong theory. In the present study, the variables that were included in the SEM analysis were primarily selected based upon findings from the correlation and regression (phase 1) analyses. For example, race and gender were initially included in the regression analysis, and were dropped from the final regression models when it was determined that they were not significant predictors of passage or failure on the Step 1 exam. In this step, it is useful to sketch a rough diagram that illustrates how you believe the variables in the model are related. If you will be using the software package AMOS (Analysis of Moment Structure) for the SEM analysis, you will need to specify the direction of the relationships between the variables by inserting arrows that illustrate these relationships. Kremelberg (2011) [13] provides excellent instructions on using AMOS to conduct a SEM analysis.
2. **Identification**: In the identification step, the model is reviewed to ensure that it is possible to estimate the measurement portion of the model. One way to accomplish this is to ensure that there are enough observable variables to serve as indicators of latent variables, which are variables that are not directly observed, but instead are constructed by combining a number of observable variables, similar to a factor when conducting factor analysis [13]. In this analysis, we did not have any latent variables, thus this step was not applicable. However, studies that make use of latent variables should keep in mind the recommendation that each latent variable needs at least two, and ideally three or four observable variables to serve as indicators of that latent variable [14].
3. **Estimation**: The model is estimated to determine the extent to which it fits the data. This involves selecting the estimation method that is most appropriate for the type of data that you are analyzing. Since one variable in this analysis was binary, we utilized the Markov Chain Monte Carlo Method. The most common estimation method, assuming the data is multivariate and normally distributed is maximum likelihood [13].
4. **Testing fit**: The goodness of fit of the model is tested. Since the Markov Chain Monte Carlo Method was used to estimate the model, the posterior predictive p-value was used to assess model fit. In our model, the value was 0.40. A posterior predictive p-value close to 0.5 indicates good model fit, and values close to 0 or 1 indicate poor model fit [15]. When testing the goodness of fit of a model in which the data is multivariate and normally distributed, measures such as the model’s chi-square or the root mean square error of approximation are most commonly used [16]. This step also involves evaluating the parameter estimates for statistical significance, appropriate directionality, and plausible magnitude in order to ensure consistency with theoretical expectations. If any issues with the parameter estimates arise at this stage, it is advisable to proceed to step five, model respecification.
5. **Respecification**: If the model does not fit the data well, then the model can be respecified, and the process starts over. For example, if the results demonstrate that one variable in the model is not related to any of the other variables in the model, it can be advantageous to remove that variable from the model, which is referred to as “trimming” the model. After trimming is completed, the process starts over with step one.

## Results

### Descriptive Statistics

The descriptive statistics associated with the analysis are presented in Table 1. The sample size of 114 students is less than our normal class size of 120 students due to a few students delaying taking the Step 1 exam, and a few cases of missing data on variables such as race and gender. Fourteen students, or approximately 12% of the sample, had an approved disability accommodation.

There was quite a bit of variation in the number of times that students utilized the services of the SASS office as indicated by the mean of 2.7, the standard deviation of 3.17, and the maximum value of 16. Not surprisingly, students with a disability accommodation used the services of the SASS office more often than students without a disability accommodation. Students with an accommodation used the services of the SASS office 4.9 times on average, as compared to 2.4 times on average for students without an accommodation. All students with an accommodation used the services of the SASS office at least twice, whereas 17% of students without an accommodation never used the services of the SASS office, and an additional 39% of students without an accommodation used the services of the SASS office only one time. Among students with an accommodation, 57.1% used the services of the SASS office three to nine times, and an additional 14.3% used the services of the SASS office ten or more times. Among students without an accommodation, 22% used the services of the SASS office three to nine times, and 6% used the services of the SASS office ten or more times.

### Phase 1: Correlation and Regression

The correlations associated with the analysis are presented in Table 2. Since the USMLE Step 1 variable is binary, the correlations associated with it must be interpreted cautiously due to the sigmoidal relationship between the variables, however they are still instructive. As the table illustrates, both performance on the course exams, and the number of consultations that students’ have with SASS are correlated with performance on the Step 1 exam. Additionally, there is a strong inverse correlation between performance on the course exams and the number of consultations that students have with SASS. For the subgroup who had disability accommodations, this was weakly correlated with the frequency of SASS consultations.

**Table 2:** Pearson correlation matrix among Step 1 outcomes, academic performance metrics, demographic variables, and student support service utilization (n =114)

	Step 1	MCAT	Undergraduate GPA	Male	Total Exam	Has disability accommodation	Number of support consultations
Step 1		.081	.173	.071	.483***	-.001	-.496***
MCAT	.081		.022	.254**	.348***	-.003	-.209*
Undergraduate GPA	.173	.022		.215*	.308***	-.102	-.028
Male	.071	.254**	.215*		.204*	-.164	-.285**
Total Exam	.483***	.348***	.308**	.204*		.001	-.534***
Has disability accommodation	-.001	-.003	-.102	-.164	.001		.265**
Number of support consultations	-.496***	-.209*	-.028	-.285**	-.534**	.265**	

*=p<.05,**=p<.01,***=p<.001

Table 3 presents the results of the logistic regression analysis. As the table illustrates, in model one, the number of consultations with accommodations office staff is a significant predictor of performance on the Step 1 exam, with an increase in the number of consultations associated with a decrease in the probability of passing the exam. Additionally, as model two illustrates, performance on the course exams is a significant predictor of performance on the Step 1 exam, as higher performance on the course exams is associated with an increase in the probability of passing the Step 1 exam. However, as model three illustrates, when both number of consultations and performance on the course exams are used as independent variables, the number of consultations with accommodations office staff is no longer a significant predictor of Step 1 performance. This is due to the strong correlation between performance on the module exams and the number of consultations with accommodations office staff, as regression analyses assume that independent variables will be uncorrelated with one another [9].

**Table 3:** Logistic regression models predicting passage of Step 1 from number of SASS consultations and total exam score (n = 114)

	Model 1			Model 2			Model 3		
	B	Exp(B)	P	B	Exp(B)	P	B	Exp(B)	P
Constant	4.306		<.001						
Number SAS Consultations	-.372	.690	<.001						
Constant				-31.328		<.001			
Total Exam Score				.046	1.048	<.001			
Constant							-24.688		.020
Number SASS Consultations							-.141	.868	.283
Total Exam Score							.038	1.039	.008
Nagelkerke R^2^	.361			.539			.558		
X^2^	17.722		<.001	27.705		<.001	28.647		<.001
-2 log likelihood	40.212			30.373			29.286		

### Phase 2: Structural Equation Modeling

**Fig. 1:**
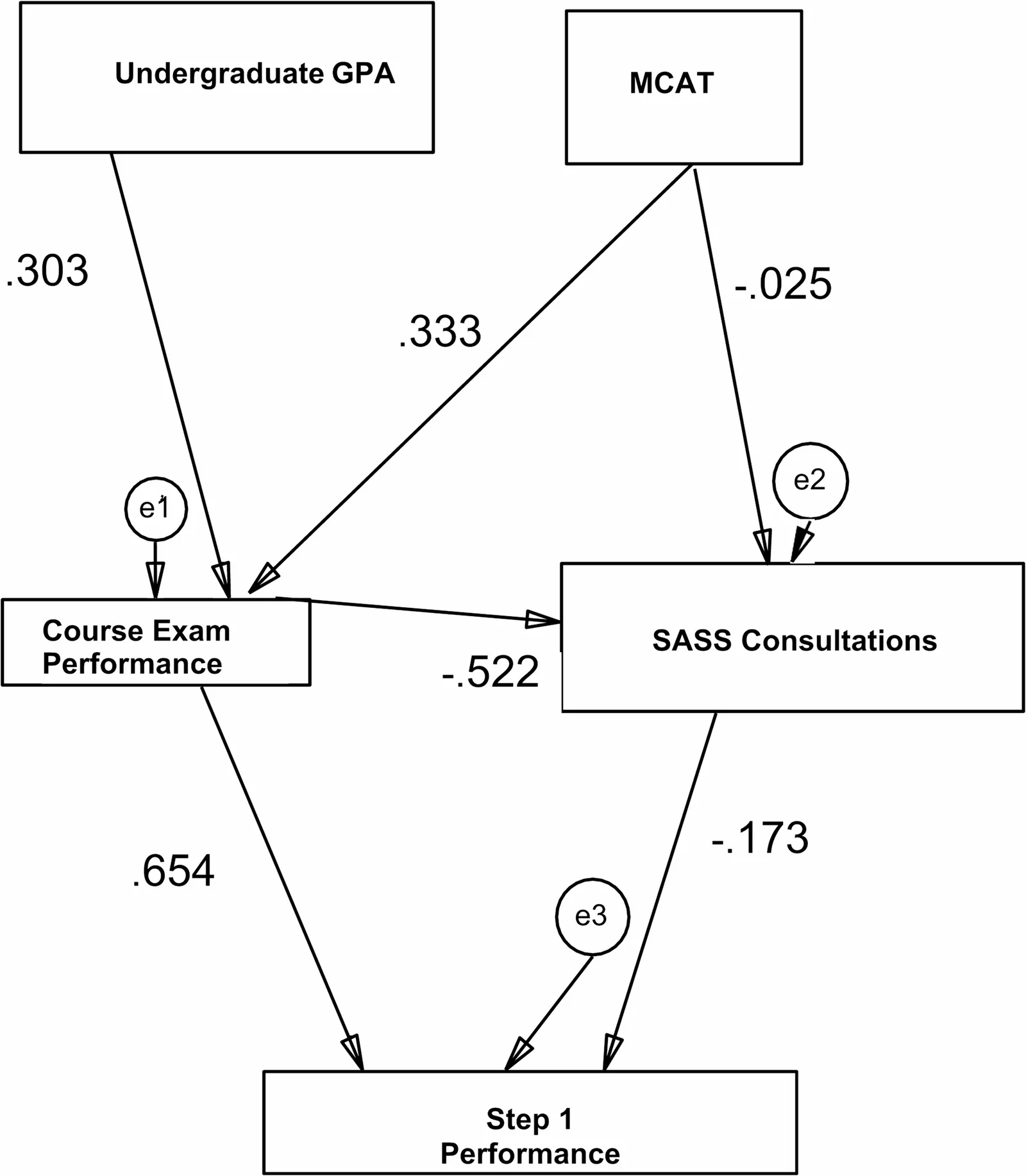
Direct and indirect relationships between undergraduate GPA, MCAT score, academic support utilization, medical school exam performance, and Step 1 outcomes (n=114). Values represent standardized path coefficients

To further explore the relationships between the variables, and to deal with the issue of multicollinearity, the data was analyzed using structural equation modeling. Because the primary outcome variable, Step 1 performance, is binary, the Markov Chain Monte Carlo analysis option was utilized in AMOS. The model that was estimated is presented in Fig. 1. The values shown are the standardized direct effects that were calculated in the analysis. These values can be interpreted similarly to standardized regression coefficients for all of the paths that do not lead directly to performance on the Step 1 exam. For the paths that do lead directly to performance on the Step 1 exam, that is the relationship between course exam performance and Step 1 performance and the relationship between number of accommodations office consultations and Step 1 performance, the coefficients have to be interpreted slightly differently. For example, the − 0.173 coefficient that represents the relationship between the number of accommodations office consultations and Step 1 performance indicates that a one standard deviation increase in the number of accommodations office consultations increases the probability of failing the Step 1 exam by approximately 0.17 standard deviations. Notably, inclusion of race, gender or disability accommodation as separate variables provided no improvement of model fit in regression or SEM analyses.

### Summary and Discussion

The first goal of this study was to quantitatively evaluate overall utilization patterns of our SASS office and the associated Step 1 outcomes. We found that academic support was widely used, but with a substantial range of engagement from zero to greater than 10 consultations per student across the preclerkship period. A central theme emerging from our data is that students who perform less well academically are more likely to seek academic support, an observation consistent with findings from a prior study conducted at two other Florida medical schools [17]. Our results further show that upstream indicators, such as MCAT score, also predict subsequent SASS usage. These findings align with large-scale MCAT validity studies demonstrating that MCAT scores and undergraduate GPA are strong predictors of medical school performance and progress [18]. Our structural equation modeling (SEM) analysis additionally revealed that students with a history of difficulty on standardized exams were more likely both to fail the Step 1 examination and to meet with SASS specialists. Recent research has also shown that low socioeconomic status (SES) predicts lower MCAT scores as well as reduced access to and awareness of effective test preparation resources [19], suggesting one direction for future work should be to examine how SES may further contribute to modeling academic support needs within our student population. At this stage, our data provides models to help predict our resource needs for academic support, though it does not provide evidence for the utility of services provided. Historically, the literature provides some evidence for efficacy of study skills interventions like our SASS office programing [20], though there is a paucity of literature demonstrating improved academic outcomes for coaching programs in general [21], and these remain important areas for future studies.

The second study goal was to determine if students with disabilities accommodations were more likely to utilize SASS or have worse USMLE Step 1 outcomes. Recalling that the general prevalence of students with disabilities in medical schools is approximately 4–5% [6], our sample with approximately 12% of students having a disability represented a large proportion of a class. We found a weak correlation between disability status and utilization of SASS services, but no correlation between disability status and Step 1 outcome, or with other scores either during or before medical school. Furthermore, when we included disability status in regression or SEM models, no improvement in model fit occurred, also indicating disability status was a minor factor. The literature generally shows that the relationships between disability status and medical school performance remains unclear, with prior studies reaching conflicting conclusions.

Some studies report no significant performance differences between students with and without disabilities on multiple choice examinations [7] or among students with dyslexia [22]. Similarly, academic progression has been found to be comparable between students who self reported a disability and those who did not [23], and disability status has not predicted success in securing a surgical specialty placement [24]. In contrast, other research indicates lower performance among students with disabilities, including reduced Step 1 [8, 25–27] and Step 2 CK scores [8, 26], lower clerkship performance [8], decreased on time graduation and first‑attempt residency match rates [25, 27], higher likelihood of taking a leave of absence [25], and lower medical school exam scores with higher exam‑failure rates [28]. In our study, however, disability status did not appear to be an important predictor of student performance; instead, upstream academic variables such as MCAT and GPA were far stronger predictors of medical school outcomes.

A possible reason for the inconsistent findings across studies examining the relationship between disability status and academic performance in medical students is that none used analytical techniques capable of handling correlated independent variables. The studies referenced above relied on methods such as logistic regression, ANOVA, chi square tests, and MANOVA, all of which are limited in their ability to address collinearity among predictors. The relationship between disability status and academic performance is an example of complex interactions. Disentangling these effects requires an analytical approach that can properly manage correlated variables, such as SEM.

Concerns such as these led to our final study goal: to evaluate the value of applying SEM to clarify direct and indirect relationships among our variables. The analysis showed that students who have more consultations with the SASS office are less likely to pass Step 1. This relationship is partly explained by the link between MCAT performance and the number of SASS consultations. The SEM results demonstrate that MCAT scores are inversely related to consultation frequency, indicating that students with a history of difficulty on standardized tests are more likely to seek support from SASS, and these students are also more likely to fail Step 1.

These findings underscore the importance of using analytical techniques such as structural equation modeling when working with datasets containing correlated independent variables. Had we relied solely on logistic regression, we would have concluded that the number of consultations with accommodations staff was not a predictor of Step 1 success once course exam performance was controlled. However, due to the strong correlation between course exam performance and consultation frequency (0.53), both variables are not independently significant predictors when both are entered into a regression model. In contrast, SEM clarifies these relationships, revealing that both course exam performance and number of consultations are significantly related to Step 1 outcomes. Our work adds to the growing body of evidence supporting SEM as a tool to avoid incorrect conclusions when collinearity confounds traditional analyses [29].

Our study points to several important directions for future research. First, replication using structural equation modeling on larger and more representative samples is needed, as the primary limitation of the current work is its focus on a single cohort from one medical school. Similar designs could also be extended to additional outcomes, including Step 2 CK performance, first-attempt residency match rates, time to degree completion, and clerkship performance. The findings additionally underscore the challenges of evaluating academic support interventions, particularly the underlying negative association between support use and outcomes that reflects pre-matriculation factors rather than the support itself. Additionally, it is possible that voluntary users of academic support services respond differently to the intervention than do mandatory users of academic support services. To more accurately assess the value added by academic support services, future work may benefit from approaches such as matched comparison groups and growth-curve modeling. Additionally, comparing voluntary and mandatory users of academic support services and how they respond, perhaps differently, to the intervention could be a fruitful avenue of future research.

## Data Availability

The data analyzed in this study are not publicly available due to institutional restrictions, and the potential of participant identification. The data are available from the corresponding author upon reasonable request with appropriate institutional permissions.

## References

[1] Hauer KE, Ciccone A, Henzel TR, Katsufrakis P, Miller SH, Norcross WA, et al. Remediation of the deficiencies of physicians across the continuum from medical school to practice: a thematic review of the literature. Acad Med. 2009;84(12):1822–32. 10.1097/ACM.0b013e3181bf3170 19940595

[2] Sandars J, Patel R, Steele H, McAreavey M. Developmental student support in undergraduate medical education: AMEE guide no. 92. Med Teach. 2014;36(12):1015–26. 10.3109/0142159X.2014.917166 25072412

[3] Frank JR, Snell LS, Cate OT, Holmboe ES, Carraccio C, Swing SR, et al. Competency-based medical education: theory to practice. Med Teach. 2010;32(8):638–45. 10.3109/0142159X.2010.501190 20662574

[4] Cutrer WB, Miller B, Pusic MV, Mejicano G, Mangrulkar RS, Gruppen LD, et al. Fostering the development of master adaptive learners: a conceptual model to guide skill acquisition in medical education. Acad Med. 2017;92(1):70–5. 10.1097/ACM.0000000000001323 27532867

[5] Lovell B. What do we know about coaching in medical education? A literature review. Med Educ. 2018;52(4):376–90. 10.1111/medu.13482 29226349

[6] Meeks LM, Case B, Plegue M, Moreland CJ, Jain S, Taylor N. National prevalence of disability and clinical accommodations in medical education. J Med Educ Curric Dev. 2020;7:2382120520965249. 10.1177/2382120520965249 PMC7592311 33178890

[7] Ricketts C, Brice J, Coombes L. Are multiple choice tests fair to medical students with specific learning disabilities? Adv Health Sci Educ Theory Pract. 2010;15(2):265–75. 10.1007/s10459-009-9197-8 19763855

[8] Teherani A, Papadakis MA. Clinical performance of medical students with protected disabilities. JAMA. 2013;310(21):2309–11. 10.1001/jama.2013.283198 24302095

[9] Kim JH. Multicollinearity and misleading statistical results. Korean J Anesthesiol. 2019;72(6):558–69. 10.4097/kja.19087 PMC6900425 31304696

[10] Violato C, Hecker KG. How to use structural equation modeling in medical education research: a brief guide. Teach Learn Med. 2007;19(4):362–71. 10.1080/10401330701542685 17935466

[11] Stoffels M, Torre DM, Sturgis P, Koster AS, Westein MP, Kusurkar RA. Steps and decisions involved when conducting structural equation modeling (SEM) analysis. Med Teach. 2023;45(12):1343–5. 10.1080/0142159X.2023.2263233 37813119

[12] Beran TN, Violato C. Structural equation modeling in medical research: a primer. BMC Res Notes. 2010;3(1):267. 10.1186/1756-0500-3-267 PMC2987867 20969789

[13] Kremelberg D. Practical statistics: a quick and easy guide to SPSS statistics, STATA, and other statistical software. Thousand Oaks (CA): SAGE; 2011.

[14] Kang H, Ahn JW. Model setting and interpretation of results in research using structural equation modeling: a checklist with guiding questions for reporting. Asian Nurs Res (Korean Soc Nurs Sci). 2021;15(3):157–62. 10.1016/j.anr.2021.06.001 34144201

[15] Cain MK, Zhang Z. Fit for a Bayesian: an evaluation of PPP and DIC for structural equation modeling. Struct Equ Model. 2019;26(1):39–50.

[16] Kline RB. Principles and practice of structural equation modeling. 4th ed. New York (NY): Guilford Press; 2023.

[17] Sookdeo SS. The relationship between the utilization of student support services and overall satisfaction in medical school [dissertation]. Tampa (FL): University of South Florida; 2016. Available from: https://digitalcommons.usf.edu/etd/6588

[18] Hanson JT, Busche K, Elks ML, Jackson-Williams LE, Liotta RA, Miller C, et al. The validity of MCAT scores in predicting students’ performance and progress in medical school: results from a multisite study. Acad Med. 2022;97(9):1374–84. 10.1097/ACM.0000000000004754 35612915

[19] Swan Sein A, McClure SC, Chanatry JA, Clinchot DM, Taylor ED, Copeland HL, et al. Examining differences in the preparation and performance of US MCAT examinees from lower-SES backgrounds: awareness, access, and action insights to narrow learning opportunity and performance gaps and promote learning for all aspiring physicians. Teach Learn Med. 2025;37(1):1–4. 10.1080/10401334.2025.2492620 40242876

[20] Van der Winston KA, Scherpbier AJ. An investigation into the design and effectiveness of a mandatory cognitive skills programme for at-risk medical students. Med Teach. 2010;32(3):236–43. 10.3109/01421590903197035 20218839

[21] Donaldson M, McBride S, Wilhelm M, Parent-Nichols J, Thomas R, Scanlon E, et al. Exploring the impact of academic coaching interventions on student outcomes in graduate healthcare and medical education: a systematic scoping review. Med Educ Online. 2025;30(1):2581671. 10.1080/10872981.2025.2581671 PMC12642885 41272926

[22] McKendree J, Snowling MJ. Examination results of medical students with dyslexia. Med Educ. 2011;45(2):176–82. 10.1111/j.1365-2923.2010.03802.x 21208263

[23] Murphy MJ, Dowell JS, Smith DT. Factors associated with declaration of disability in medical students and junior doctors, and the association of declared disability with academic performance: observational study using data from the UK Medical Education Database, 2002–2018 (UKMED54). BMJ Open. 2022;12(4):e059179. 10.1136/bmjopen-2021-059179 PMC8977810 35361617

[24] Ellis R, Al-Tawarah Y, Brennan PA, Lee AJ, Hines J, Scrimgeour DS, et al. The impact of disability on recruitment to higher surgical specialty training: a retrospective cohort study. Surgeon. 2024;22(6):344–51. 10.1016/j.surge.2024.07.006 39084924

[25] Meeks LM, Plegue M, Swenor BK, Moreland CJ, Jain S, Grabowski CJ, et al. The performance and trajectory of medical students with disabilities: results from the pathways project. Acad Med. 2021;96(Suppl 1):S209–10. 10.1097/ACM.0000000000004271 34705710

[26] Purkiss J, Plegue M, Grabowski CJ, Kim MH, Jain S, Henderson MC, et al. Examination of medical college admission test scores and US medical licensing examination step 1 and step 2 clinical knowledge scores among students with disabilities. JAMA Netw Open. 2021;4(5):e2110914. 10.1001/jamanetworkopen.2021.10914 PMC8140368 34019088

[27] Meeks LM, Plegue M, Swenor BK, Moreland CJ, Jain S, Grabowski CJ, et al. The performance and trajectory of medical students with disabilities: results from a multisite, multicohort study. Acad Med. 2022;97(3):389–97. 10.1097/ACM.0000000000004510 PMC8855952 34817411

[28] Revell K, Nolan H. Do medical students with a disability experience adverse educational outcomes on UK medical courses? Med Teach. 2023;45(4):388–94. 10.1080/0142159X.2022.2136518 36308742

[29] Rajdeep G, Cote JA, Baumgartner H. Multicollinearity and measurement error in structural equation models: implications for theory testing. Mark Sci. 2004;23(4):519–29.

